# Preferred resting surfaces of dominant malaria vectors inside different house types in rural south-eastern Tanzania

**DOI:** 10.1186/s12936-020-3108-0

**Published:** 2020-01-15

**Authors:** Betwel J. Msugupakulya, Emmanuel W. Kaindoa, Halfan S. Ngowo, Japhet M. Kihonda, Najat F. Kahamba, Dickson S. Msaky, Damaris Matoke-Muhia, Patrick K. Tungu, Fredros O. Okumu

**Affiliations:** 1 Environmental Health and Ecological Sciences Department, Ifakara Health Institute, P. O. Box 53, Ifakara, Tanzania; 2 School of Life Science and Bioengineering, The Nelson Mandela African Institution of Science and Technology, P. O. Box 447, Arusha, Tanzania; 3 School of Public Health, Faculty of Health Sciences, University of the Witwatersrand, Johannesburg, South Africa; 4 Institute of Biodiversity, Animal Health and Comparative Medicine, University of Glasgow, Glasgow, G12 8QQ UK; 5 Centre for Biotechnology Research and Development, Kenya Medical Research Institute, Nairobi, Kenya; 6 Amani Medical Research Centre, National Institute of Medical Research, Muheza, Tanzania

**Keywords:** Indoor residual spraying, Contact insecticides, House screening, Malaria vectors, *An. funestus*, *An. arabiensis*, Ifakara Health Institute, Indoor resting behaviours

## Abstract

**Background:**

Malaria control in Africa relies extensively on indoor residual spraying (IRS) and insecticide-treated nets (ITNs). IRS typically targets mosquitoes resting on walls, and in few cases, roofs and ceilings, using contact insecticides. Unfortunately, little attention is paid to where malaria vectors actually rest indoors, and how such knowledge could be used to improve IRS. This study investigated preferred resting surfaces of two major malaria vectors, *Anopheles funestus* and *Anopheles arabiensis*, inside four common house types in rural south-eastern Tanzania.

**Methods:**

The assessment was done inside 80 houses including: 20 with thatched roofs and mud walls, 20 with thatched roofs and un-plastered brick walls, 20 with metal roofs and un-plastered brick walls, and 20 with metal roofs and plastered brick walls, across four villages. In each house, resting mosquitoes were sampled in mornings (6 a.m.–8 a.m.), evenings (6 p.m.–8 p.m.) and at night (11 p.m.–12.00 a.m.) using Prokopack aspirators from multiple surfaces (walls, undersides of roofs, floors, furniture, utensils, clothing, curtains and bed nets).

**Results:**

Overall, only 26% of *An. funestus* and 18% of *An. arabiensis* were found on walls. In grass-thatched houses, 33–55% of *An. funestus* and 43–50% of *An. arabiensis* rested under roofs, while in metal-roofed houses, only 16–20% of *An. funestus* and 8–30% of *An. arabiensis* rested under roofs. Considering all data together, approximately 40% of mosquitoes rested on surfaces not typically targeted by IRS, i.e. floors, furniture, utensils, clothing and bed nets. These proportions were particularly high in metal-roofed houses (47–53% of *An. funestus*; 60–66% of *An. arabiensis*).

**Conclusion:**

While IRS typically uses contact insecticides to target adult mosquitoes on walls, and occasionally roofs and ceilings, significant proportions of vectors rest on surfaces not usually sprayed. This gap exceeds one-third of malaria mosquitoes in grass-thatched houses, and can reach two-thirds in metal-roofed houses. Where field operations exclude roofs during IRS, the gaps can be much greater. In conclusion, there is need for locally-obtained data on mosquito resting behaviours and how these influence the overall impact and costs of IRS. This study also emphasizes the need for alternative approaches, e.g. house screening, which broadly tackle mosquitoes beyond areas reachable by IRS and ITNs.

## Background

Malaria control efforts have yielded significant success in recent decades, resulting in decline in number malaria cases from 239 million in 2010 to 219 million in 2017 [1]. The most widely used interventions, namely insecticide-treated nets (ITNs), indoor residual spraying (IRS) and artemisinin-based combination therapy (ACT) are credited with 663 million clinical cases of malaria averted between 2000 and 2015 [2]. In Tanzania, the impact of these interventions has been demonstrated by multiple investigators [3–6], as well as national surveys, which show significant overall reduction in burden [7]. Despite these gains, there is also evidence that the anti-malaria progress is levelling off and that the gains may be lost [1]. Between 2015 and 2017, continued utilization of the core interventions led to no significant declines in malaria at global scale [1].

To rejuvenate the malaria fight, several countries have set ambitious goals in line with the WHO Global Technical Strategy for Malaria Elimination [8], and more recently, the High Burden to High Impact initiative which targets the ten most malarious countries in Africa, plus India [9]. The new initiatives are expected to be much more aggressive and country-led but involving multiple partners. However, similar to previous efforts, these efforts are primarily reliant on ITNs [now long-lasting insecticide-treated nets (LLINs)], IRS and effective case management [1]. Despite proven effectiveness of the vector control interventions, LLINs and IRS are negatively affected by insecticide resistance [10, 11], increasing outdoor-biting [12–14], high costs and the sub-optimal coverage and usage at community and household level. Resistance is often associated with exposure of vectors to insecticides used in agriculture [15] and public health [16, 17], and the indoor interventions may also induce shifts in vector biting and resting behaviours [18–20].

IRS is one of the oldest malaria interventions and was the most important component of the initial attempts at global malaria eradication in 1950s and 1960s [21, 22]. It involves applying insecticides to kill mosquitoes resting on interior walls of houses [23]. In Tanzania, it has been used intermittently since the 1960s [24], and is currently deployed in selected districts mostly in the northern regions where malaria burden remains very high [25, 26]. Across Africa, IRS is mostly promoted by the US Presidents Malaria Initiative (PMI), and currently covers 14 countries in Africa [27, 28]. According to the 2015 analysis by Bhatt et al. [2], IRS alone contributed to 10% of averted clinical malaria cases in Africa between 2000 and 2015. To counter the growing challenge of insecticide resistance [29], most countries have switched from using pyrethroids, and now rely mostly on organophosphates or carbamates, as well as some new insecticide classes such as neonicotinoids, which were recently introduced [30]. There have also been calls to introduce bed nets with multiple active ingredients or synergists as a way to tackle resistance [31–33].

While much of the focus is paid to finding new chemical actives and combinations, considerably less attention is paid to how malaria mosquitoes actually respond to the indoor interventions such as IRS and LLINs. This is despite the changing housing designs and structures across Africa [34], and the demonstrated impact of housing on vector densities and malaria transmission [35–38]. Instead indoor interventions still primarily rely on historical evidence of mosquito indoor resting habits [39, 40], which are now due for update in light of modern transformations [34]. A study from early 1960s in Tanzania assessed distribution of malaria vectors on sprayable surfaces inside houses compared to household possessions usually removed during IRS [39]. It was observed that less than 20% of mosquitoes rested on the possessions, and that of the remaining, sprayable surfaces, the resting populations were evenly divided between substrates [39]. In a separate study in mud huts in northern Tanzania, 56% to 70% of all resting mosquitoes were found on the walls or hanging articles, while the remaining 30% to 40% were on the underside of the roofs [40].

Other than these early studies, such investigations have become rare, yet it is likely that mosquito behaviours and survival inside houses could change with the ongoing improvements. For example, a recent study in the Gambia demonstrated that reduced mosquito survival in metal-roofed houses may lower malaria transmission [41]. Elsewhere in East Africa, it was shown that despite higher temperatures inside houses with corrugated iron roofs, survival of mosquitoes resting indoors was same as in grass thatched houses [42].

It is, therefore, crucial to understand resting behaviours of the major malaria vectors inside houses and how much they can be affected by key indoor interventions. This way, effectiveness of techniques such as IRS can be improved, and their limitations determined. This study therefore investigated the resting behaviours of two major malaria mosquitoes (*An. funestus* and *An. arabiensis*) inside typical house types in rural south-eastern Tanzania. In this area, most malaria infections are mediated by *An. funestus*, even though *An. arabiensis* remains abundant as well [43, 44].

## Methods

### Study area

**Fig. 1:**
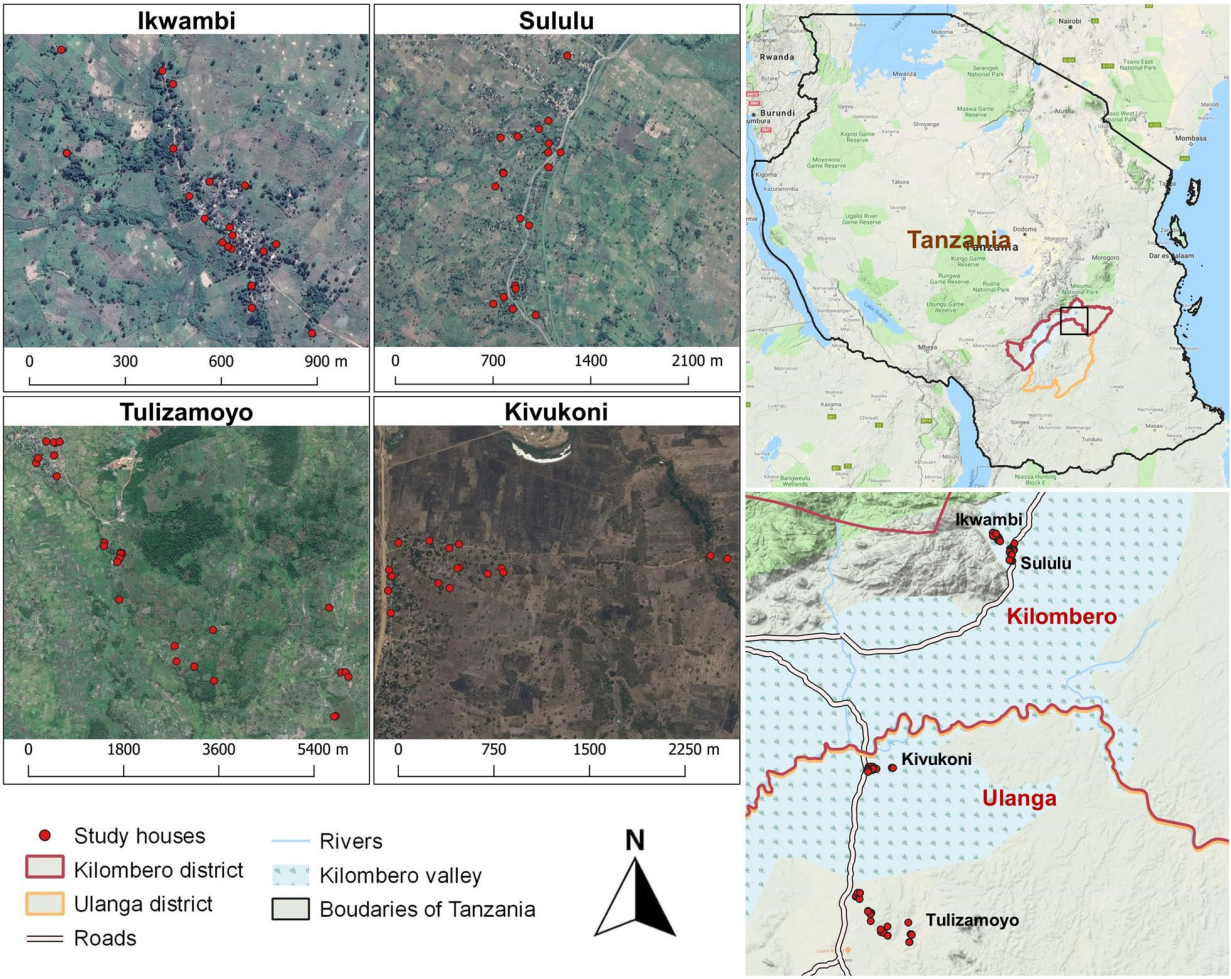
Map showing study villages and study households in both Kilombero and Ulanga districts, south-eastern Tanzania. Indoor-resting mosquitoes were collected multiple times from each household during the study period

The study was conducted in four villages across Ulanga and Kilombero districts in south-eastern Tanzania (Fig. 1). These included, Kivukoni (− 8.2021, 36.6961) and Tulizamoyo (− 8.3669, 36.7336) in Ulanga district, and Sululu (− 7.9973, 36.8317) and Ikwambi (− 7.9833, 36.8184) in Kilombero district. The area is within a low-lying river valley extending 250 km long and up to 65 km wide, interspersed with villages and farmlands. It has two rainy seasons, short rains between November and December and long rains between March and May, while between rainy seasons spans two dry seasons. Annual rainfall and temperatures vary from 1200 to 1800 mm, and 16 °C to 32 °C, respectively [45]. Residents are mostly subsistence farmers, though some are also fishermen or owned small businesses.

During this study, typical house types in the villages were either thatch-roofed or metal-roofed (with corrugated iron sheets), and had either mud walls or brick walls, which were sometime plastered with concrete. Primary malaria vectors in this region are *An. funestus* and *An. arabiensis*, with *An. funestus* contributing more than 80% of current malaria transmission [44]. *Culex pipiens* are nuisance biters contributing 79% of all indoor biting risk [46].

### Selection and characterization of study houses

**Fig. 2:**
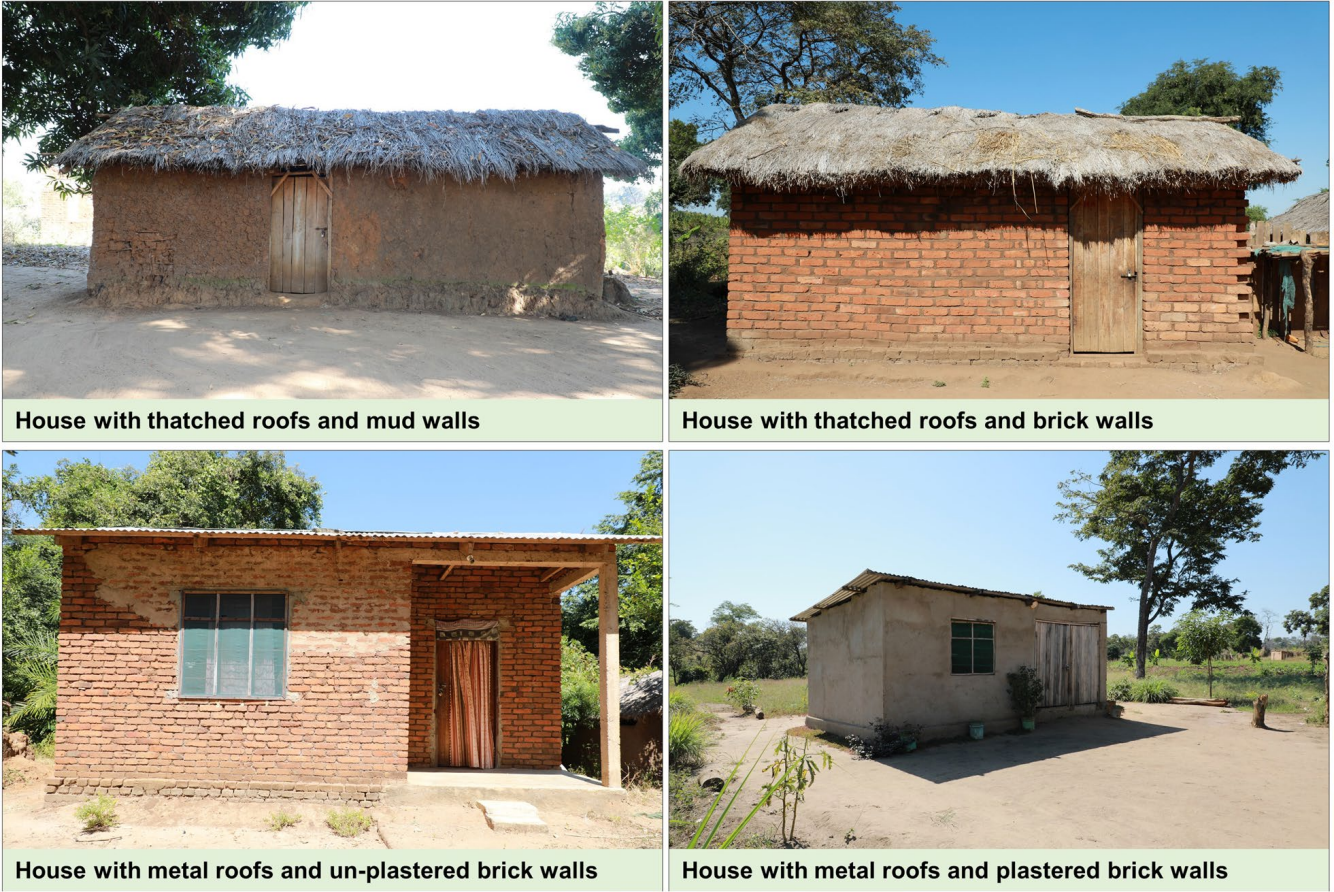
Typical house types in the study villages in rural south-eastern Tanzania. The pictures depict only outside views of the houses, and does not show actual concrete plastering of some brick walls. These four are used as representative of the different house types, but the actual sizes and shapes of individual houses was varied

Field collection of resting mosquitoes was done inside human-occupied houses, ensuring to cover the main house types. Candidate houses were selected based on construction materials for walls (mud or bricks, with or without concrete plastering) and roofs (metal or thatch). This resulted in four classes of houses (Fig. 2) commonly found in the study area, namely: (i) houses with thatched roofs and mud walls, (ii) houses with thatched roofs and brick walls (none of these houses had plastered walls), (iii) houses with metal roofs and un-plastered brick walls, and (iv) houses with metal roofs and plastered brick walls. Ceilings were uncommon and therefore excluded in this survey. All individual houses were also geo-referenced, then characterized by other attributes, namely: (a) whether eave gaps were open or closed, (b) number of rooms in the house, (c) height of walls and (d) maximum daily temperatures (°C), recorded using Tinytag^®^ data loggers (Gemini, UK) suspended from the roofs, more than 1 m from the floor.

Prior to commencement of mosquito collections, 20 houses were purposively selected in each of the four villages upon consent by household heads. These included five houses per house type.

### Collection of mosquitoes resting on different surfaces inside the houses

Potential mosquito resting places were identified to include: (a) walls, (b) roofs (underside of the roofs) and (c) other surfaces such as floor, clothing, bed nets and other household items. The household items were varied but generally included furniture such as beds, tables, chairs, cupboards, wood blocks, other household items such as bicycles, and utensils, wash basins, water containers, clay pots and cooking pans. The clothing included hanging garments, curtains, sacks and bags. Actual mosquito collections were done using Prokopack aspirator [47], by trained technicians. Collections involved hovering the aspirator systematically over the surfaces and collecting all mosquitoes. Lighting was provided using hand-held flash lights. The sequence of collection between resting surfaces in each room was changed to minimize sampling biases. The collections were done for 5 days each week in each village, visiting 2–4 houses per day. Initially the collections were done between 6 a.m. and 12 p.m, from January 2019 to May 2019. Then from May to July 2019, the collections were done three times a day [in the morning (between 7:00 a.m. and 8:30 a.m.), evening (between 6:00 p.m. and 8:00 p.m.) and at night (between 12:00 a.m. and 2:00 a.m.)], to minimize variations associated with mosquitoes moving between different resting surfaces within the houses. Unlike the other collections done by trained technicians, the late evening and late-night collections were done by trained household members to avoid intrusion of their privacy.

In total, there were 277 house visits for indoor resting mosquito collections, including 76 visits to houses with thatched roofs and mud walls, 70 to houses with thatched roofs and brick walls, 70 to houses with corrugated iron roofs and un-plastered brick walls, and 61 visits to houses with corrugated iron roofs and plastered brick walls.

### Morphological identification and processing of collected mosquitoes

Mosquitoes collected from each of the resting surfaces were placed in separate disposable cups and labelled appropriately. They were sorted by sex and taxa, then all *Anopheles* sorted and identified using the morphological keys [48]. Physiological status of each female *Anopheles* was determined as unfed, partly fed, fully fed, gravid or semi gravid. All records were kept by house, surface, house type and village.

### Identification of sibling species of malaria vectors, blood meal analysis and detection of *Plasmodium falciparum* sporozoites in the mosquitoes

The field-collected mosquitoes were packed individually in 1.5 ml microcentrifuge tubes (BioPointe Scientific^®^) containing silica plugged with cotton wool. Sub-samples of *An. funestus* sensu lato (s.l.) and *Anopheles gambiae s.l.* females were further analysed for sibling species, *Plasmodium falciparum* sporozoites and blood meal sources (if the mosquitoes were blood-fed). Sibling species identification for *An. funestus* s.l. and *An. gambiae* s.l. was done using PCR protocols originally developed by Koekemoer et al. [49] and Scott et al. [50] respectively. Blood meal analysis was done using ELISA tests [51], and parasite infections detected by screening for the *P. falciparum* circumsporozoite proteins in salivary glands of the adult females [52]. Heat-labile non-*P. falciparum* were eliminated by boiling the ELISA lysates at 100 °C for 10 min to remove false positives [53].

### Determination of physiological ages of mosquitoes

Parity of mosquitoes was approximated following procedure described by Detinova [54] as a proxy of physiological age of mosquitoes. A subsample of non-blood fed, *An. funestus* and *An. arabiensis*, were first immobilized in a refrigerator. Under stereo microscope abdomens of anesthetized mosquitoes were dissected to extract ovaries. Ovaries were examined under compound microscope to determine whether mosquitoes had laid eggs or not.

### Data analysis

Data analysis was done using open source statistical software, R version 3.6.0 [55]. Generalized linear mixed effects models (GLMM) were built using functions within the *lme4* package [56] to assess: (i) preferences of mosquitoes (*An. funestus*, *An. arabiensis* and *Culex*) for different resting surfaces and (ii) relationships between various household risk factors and number of mosquitoes caught on different surfaces. Initially, the number of female mosquitoes of each species was modelled as a response variable against resting surfaces as a fixed factor. Since walls are typically the main target for insecticide spraying, they were used as reference against which other surfaces were compared.

To assess relationships between household risk factors and mosquitoes resting on different surfaces, the number of mosquitoes caught from each surface was modelled as function of: (i) roof type, (ii) wall type, (iii) whether interior walls were plastered with cement or not, (iv) eave gaps, (v) number of rooms, (vi) wall height and (vii) daily maximum temperatures inside the houses.

In all models, households nested within villages and sampling days were used as random terms, to capture unexplained variations, and account for pseudo-replication. Poison distribution was used when fitting GLMM models, except when overdispersion was detected, in which cases, negative binomial distribution was used instead. The best fitting models were selected using Akaike Information Criterion (AIC) [57], and results presented as relative rate ratios (RR) at 95% confidence intervals. In addition, the *dabestr* package for estimation statistics [58], was used to depict effect sizes of differences in mean numbers (at 95% confidence intervals) of mosquitoes collected on different resting surfaces relative to walls.

## Results

### Descriptive summary of mosquitoes caught in the surveys

A total of 17,870 female mosquitoes were collected, of which 31.1% (n = 5564) were *Anopheles* mosquitoes and 68.9% (n = 12,306) were culicines. Among *Anopheles* mosquitoes, 81.5% (n = 4535) were *An. funestus* s.l., 17.6% (n = 977) were *An. arabiensis* and 0.9% (n = 52) were other *Anopheles* species including *Anopheles coustani* and *Anopheles pharoensis*. The majority of *An. funestus* (72.4%), *An. arabiensis* (87.8%) and *Culex* (58.0%) were collected in thatch-roofed houses.

### Resting preferences of mosquitoes inside the houses

**Fig. 3:**
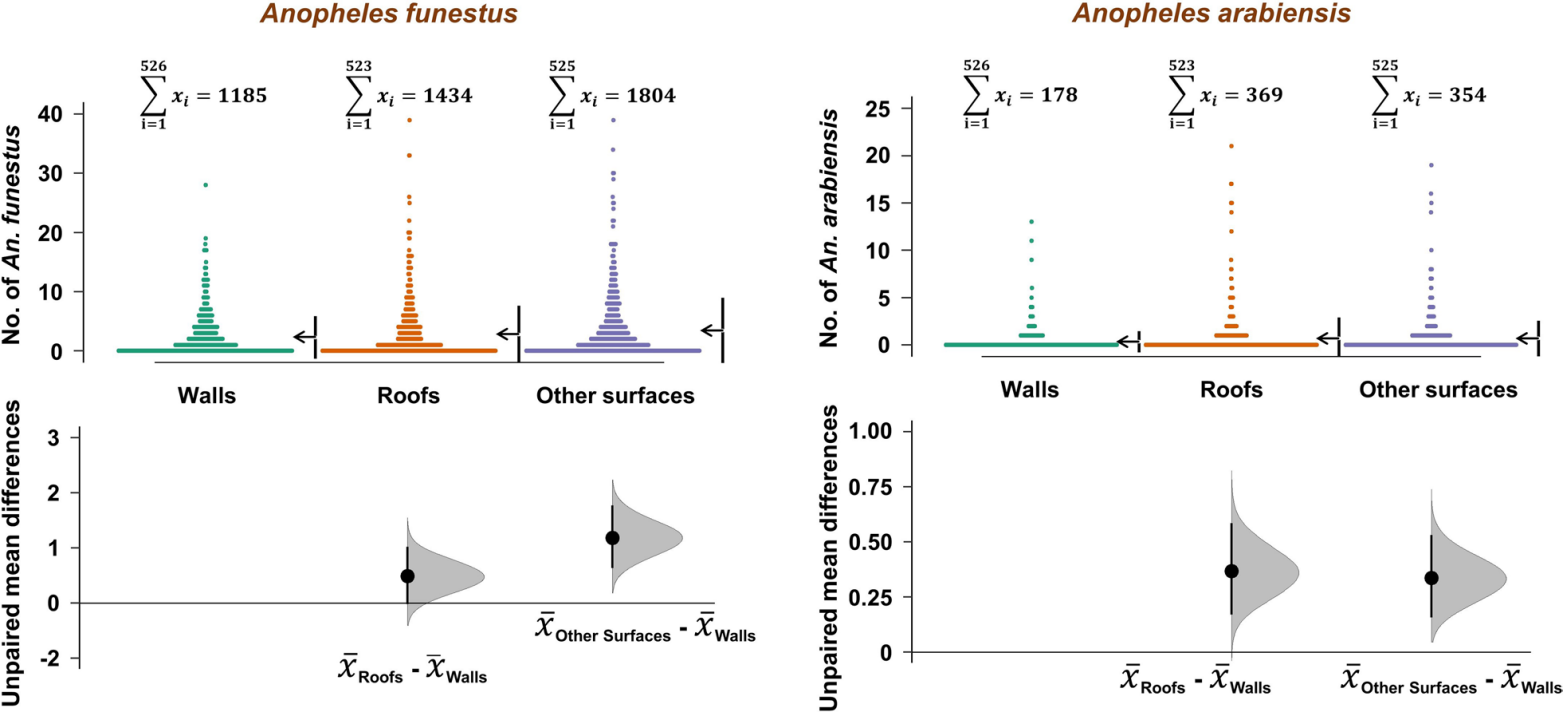
Overall nightly densities of malaria vectors *Anopheles funestus* and *Anopheles arabiensis*, from different resting surfaces inside the houses. This data is aggregated for all house types in the study area. Estimation plots are provided to depict distribution of residuals between collections from roofs or other surfaces, and the collections on walls. The number of mosquitoes resting on either roofs or other surfaces are considered significantly higher or lower, based on how far the means of the residuals are above or below the reference line

There was an uneven distribution of mosquitoes between the four house types and between the different resting surfaces (Tables 1, 2 and Fig. 3). Only 26.1% of *An. funestus,* 18.2% of *An. arabiensis* and 27.9% of *Culex* mosquitoes rested on walls. Proportions resting on the undersides of the roofs included 32.9% of *An. funestus*, 42% of *An. arabiensis* and 33.6% of *Culex* mosquitoes. Surprisingly, as many as 41% *An. funestus*, 40% of *An. arabiensis* and 39% of *Culex* mosquitoes rested on surfaces other than either the walls or roofs, i.e. surfaces that are not typically sprayed during IRS. The actual distribution of the two malaria vector species and the *Culex* mosquitoes also depended on house construction materials. Nearly 80% of *An. funestus* and *An. arabiensis* were collected in grass-thatched houses and the remainder in the metal-roofed houses. However, once inside the houses, proportions resting under the roof surfaces was generally lower in metal-roofed houses (*An. funestus*, 16.0–20.0%; *An. arabiensis*, 7.6–30.0%) than in grass-thatched houses (*An. funestus*, 32.5–55.2%; *An. arabiensis*, 43.1–49.8%). The proportions of mosquitoes resting on surfaces not typically sprayed were approximately one-third in grass-thatched houses, and between one half and two-third in metal-roofed houses. Full details including distribution of *Culex* mosquitoes are shown in Table 1.

**Table 1:** Numbers and percentages of mosquitoes of different species collected from different surfaces inside houses of different types in Ulanga and Kilombero districts, south-eastern Tanzania

Species	Resting surfaces inside houses	Thatched roofs and mud walls	Thatched roofs and brick walls	Metal roofs and un-plastered brick walls	Metal roofs and plastered brick walls	Totals
		n (%)	n (%)	n (%)	n (%)	N (%)
*Anopheles funestus*	Walls	168 (17.9)	573 (24.5)	385 (37.1)	59 (27.4)	1185 (26.1)
	Roofs	519 (55.2)	762 (32.5)	166 (16.0)	43 (20.0)	1490 (32.9)
	Other surfaces	253 (26.9)	1008 (43.0)	486 (46.9)	113 (52.6)	1860 (41.0)
	Total	940	2343	1037	215	4535
*Anopheles arabiensis*	Walls	111 (21.0)	42 (12.7)	21 (26.6)	4 (10.0)	178 (18.2)
	Roofs	227 (43.1)	165 (49.8)	6 (7.6)	12 (30.0)	410 (42.0)
	Other surfaces	189 (35.9)	124 (37.5)	52 (65.8)	24 (60.0)	389 (39.8)
	Total	527	331	79	40	977
*Culex mosquitoes*	Walls	1089 (25.2)	700 (25.4)	683 (32.2)	929 (31.1)	3401 (27.9)
	Roofs	1926 (44.6)	1352 (49.0)	389 (18.3)	431 (14.4)	4098 (33.6)
	Other surfaces	1300 (30.1)	707 (25.6)	1051 (49.5)	1630 (54.5)	4688 (38.5)
	Total	4315	2759	2123	2990	12,187

**Table 2:** Summary statistics for comparison of the number of mosquitoes of different species collected from walls, roofs and other surfaces inside the different house types, in Ulanga and Kilombero districts, south-eastern Tanzania

House type	Resting surfaces				Mosquito species					
		*Anopheles funestus*			*Anopheles arabiensis*			*Culex* mosquitoes		
		Mean (95% CI)	RR (95% CI)	p value	Mean (95% CI)	RR (95% CI)	p value	Mean (95% CI)	RR (95% CI)	p value
Thatched roofs and mud walls	Walls	0.26 (0.08–0.90)	1.00		0.12 (0.05–0.29)	1.00		2.02 (0.70–5.80)	1.00	
	Roofs	0.71 (0.21–2.43)	2.72 (2.11–3.50)	0.001	0.23 (0.09–0.55)	1.93 (1.33–2.79)	0.001	3.56 (1.24–10.24)	1.77 (1.50–2.08)	0.001
	Other surfaces	0.39 (0.11–1.34)	1.49 (1.14–1.94)	0.003	0.21 (0.08–0.50)	1.73 (1.20–2.51)	0.004	2.63 (0.91–7.57)	1.31 (1.11–1.54)	0.001
Thatched roofs and brick walls	Walls	0.63 (0.14–2.76)	1.00		0.15 (0.07–0.29)	1.00		3.58 (1.58–8.10)	1.00	
	Roofs	0.73 (0.17–3.23)	1.17 (0.94–1.45)	0.170	0.34 (0.18–0.64)	2.27 (2.27–2.28)	0.001	6.16 (2.73–13.92)	1.72 (1.37–2.16)	0.001
	Other surfaces	1.02 (0.23–4.49)	1.63 (1.31–2.02)	0.001	0.29 (0.15–0.56)	1.97 (1.96–1.97)	0.001	4.16 (1.84–9.39)	1.16 (0.92–1.47)	0.210
Metal roofs and un-plastered brick walls	Walls	0.75 (0.27–2.11)	1.00		0.06 (0.02–0.16)	1.00		3.08 (1.16–8.19)	1.00	
	Roofs	0.32 (0.11–0.90)	0.42 (0.32–0.57)	0.001	0.02 (0.01–0.06)	0.29 (0.12–0.71)	0.007	1.56 (0.58–4.18)	0.51 (0.37–0.69)	0.001
	Other surfaces	0.94 (0.33–2.63)	1.25 (0.96–1.63)	0.100	0.10 (0.04–0.25)	1.60 (0.93–2.78)	0.090	4.62 (1.74–12.2)	1.50 (1.15–1.97)	0.003
Metal roofs and plastered brick walls	Walls	0.15 (0.03–0.64)	1.00		–	–	–	1.75 (0.48–6.44)	1.00	
	Roofs	0.11 (0.02–0.47)	0.73 (0.50–1.07)	0.110	–	–	–	1.12 (0.30–4.11)	0.64 (0.48–0.83)	0.001
	Other surfaces	0.28 (0.06–1.22)	1.92 (1.41–2.59)	0.001	–	–	–	3.48 (0.95–12.70)	1.98 (1.56–2.52)	0.001

In the statistical analyses, the wall is considered reference, against which other surfaces are compared. The tables include model estimated mean catches per collection event per house per night. There were three collection events each night

Very few *An. arabiensis* were caught in houses with metal roofs and plastered brick walls, it was not possible to fit GLMM model to *An. arabiensis* data

Table 2 shows the extent to which mosquitoes preferred roofs and other internal house surfaces, compared to walls. Generally, the proportion of mosquitoes resting on non-sprayed surfaces (other surfaces) was always higher than proportions resting on walls regardless of house type. However, proportions resting on roofs was higher than on walls for grass-thatched houses, but lower for metal-roofed houses (Table 2).

**Fig. 4:**
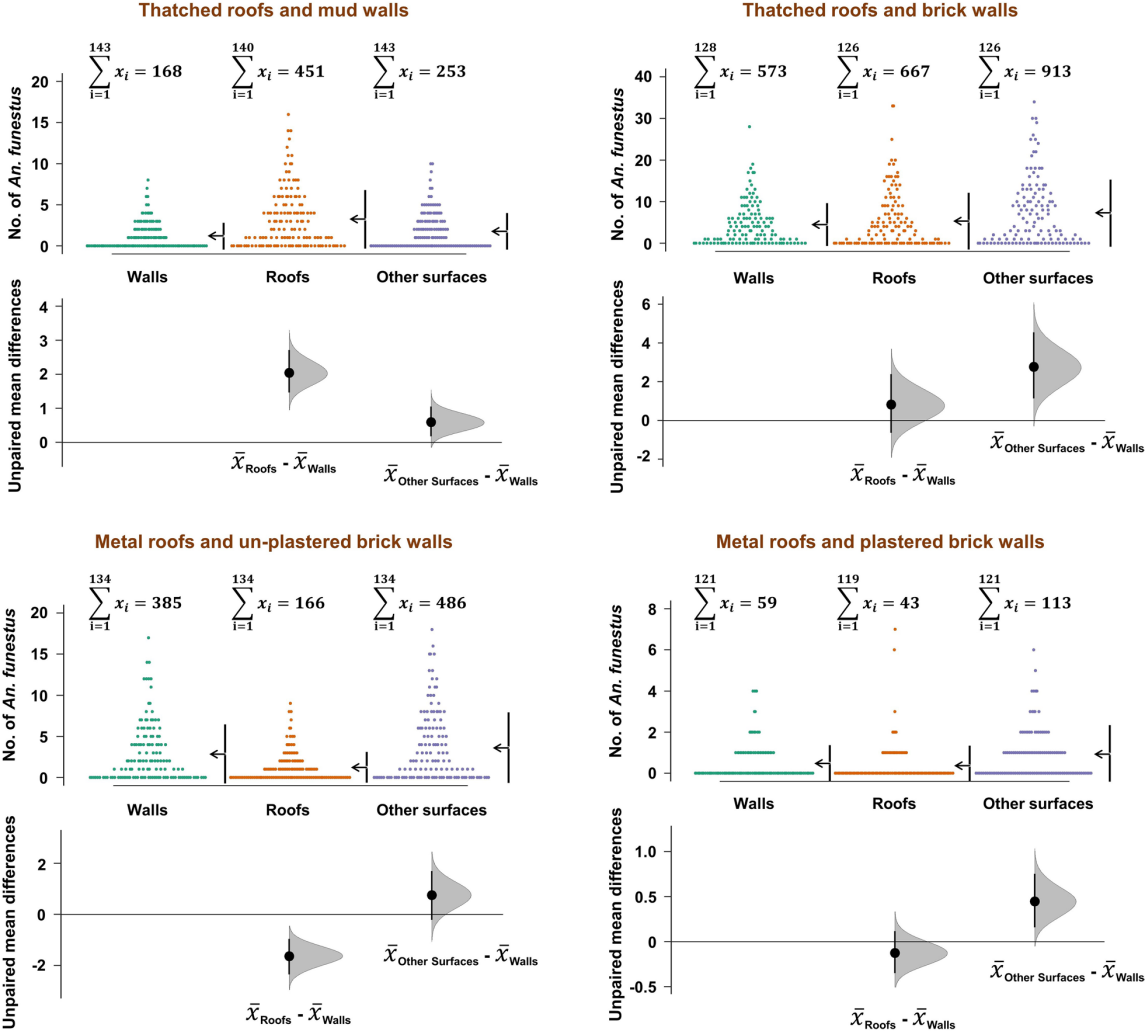
Comparison of *Anopheles funestus* densities on different resting surfaces in different house types. Estimation plots are provided to depict distribution of residuals between collections from roofs or other surfaces, and the collections on walls. Numbers of mosquitoes resting on either roofs or other surfaces are considered significantly higher or lower based on how far the means of the residuals are above or below the reference line

**Fig. 5:**
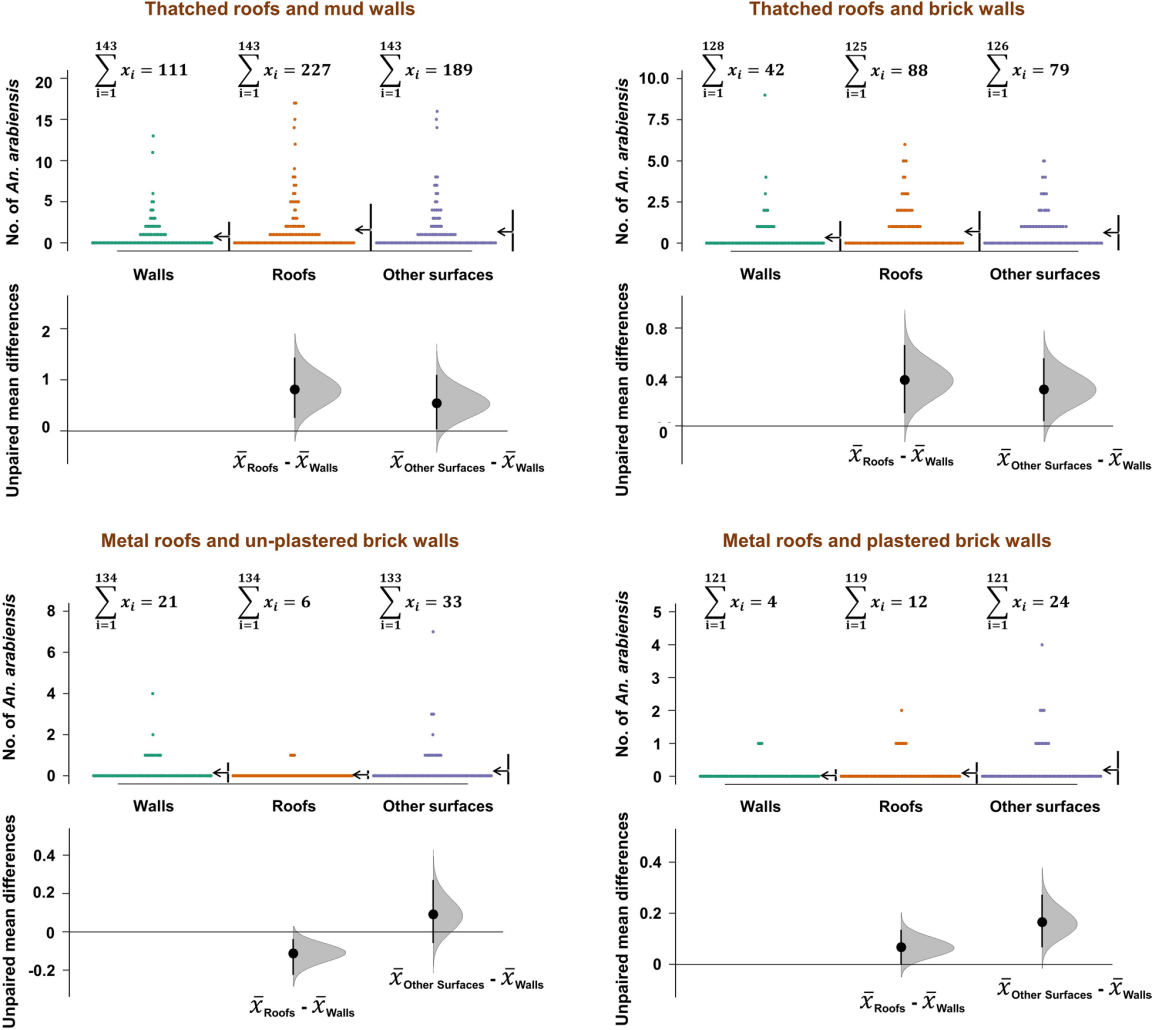
Comparison of *Anopheles arabiensis* densities on different resting surfaces in different house types. Estimation plots are provided to depict distribution of residuals between collections from roofs or other surfaces, and the collections on walls. Numbers of mosquitoes resting on either roofs or other surfaces are considered significantly higher or lower based on how far the means of the residuals are above or below the reference line

When the data was examined for different house types, it became clear that wall surfaces, at best had only one-third of mosquitoes resting. Depending on house construction materials, proportions of mosquitoes resting on roofs and other surfaces was often higher than on walls, except in metal-roofed houses, where walls tended to harbour more mosquitoes (Figs. 4 and 5). Data for *Culex* mosquitoes is shown in Tables 1 and 2, and in Additional file 1. When the other surfaces were examined in detail, it was observed that significant proportions of mosquitoes on these surfaces were resting on bed nets, floors, and on furniture, but also on hanging clothes. Full details are provided in Table 3.

**Table 3:** Numbers and percentages of mosquitoes of different species collected from surfaces typically not targeted by IRS inside houses of different types, in Ulanga and Kilombero districts, south-eastern Tanzania

Species	Resting surfaces inside houses	Thatched roofs and mud walls	Thatched roofs and brick walls	Metal roofs and un-plastered brick walls	Metal roofs and plastered brick walls	Totals
		n (%)	n (%)	n (%)	n (%)	N (%)
*Anopheles funestus*	Floor	129 (12.8)	29 (25.7)	125 (25.7)	48 (19.0)	331 (17.8)
	Furniture	186 (18.5)	18 (15.9)	80 (16.5)	87 (34.4)	371 (19.9)
	Bed nets	587 (58.2)	25 (22.1)	79 (16.3)	59 (23.3)	750 (40.3)
	Clothes	74 (7.3)	31 (27.4)	134 (27.6)	32 (12.6)	271 (14.6)
	Utensils	32 (3.2)	10 (8.8)	68 (14.0)	27 (10.7)	137 (7.4)
	Total	1008	113	486	253	1860
*Anopheles arabiensis*	Floor	25 (20.2)	8 (33.3)	16 (30.8)	36 (19.0)	85 (21.9)
	Furniture	18 (14.5)	7 (29.2)	6 (11.5)	54 (28.6)	85 (21.9)
	Bed nets	63 (50.8)	1 (4.2)	15 (28.8)	24 (12.7)	103 (26.5)
	Clothes	9 (7.3)	3 (12.5)	9 (17.3)	41 (21.7)	62 (15.9)
	Utensils	9 (7.3)	5 (20.8)	6 (11.5)	34 (18.0)	54 (13.9)
	Total	124	24	52	189	389
*Culex mosquitoes*	Floor	209 (29.6)	458 (28.1)	275 (26.2)	261 (20.1)	1203 (25.7)
	Furniture	189 (26.7)	470 (28.8)	191 (18.2)	461 (35.5)	1311 (28.0)
	Bed nets	100 (14.1)	123 (7.5)	236 (22.5)	100 (7.7)	559 (11.9)
	Clothes	125 (17.7)	368 (22.6)	175 (16.7)	236 (18.2)	904 (19.3)
	Utensils	84 (11.9)	211 (12.9)	174 (16.6)	242 (18.6)	711 (15.2)
	Total	707	1630	1051	1300	4688

When interaction was assessed between time of collection and number of resting mosquitoes. Significant interaction was observed between number of *An. funestus* resting on roof in the evening. There was no any other significant interaction between time of collection and number of resting mosquitoes (Additional file 1: Table S1).

### Effects of household variables on preferences of mosquitoes for different resting surfaces inside houses

Associations between household risk factors and proportions of mosquitoes in different resting surfaces are summarized in Table 4. Generally, compared to metal-roofed houses, grass-thatched houses had more mosquitoes of all taxa, and on all surfaces. In most cases, the number of mosquitoes in grass-thatched houses was more than double that in metal-roofed houses. Compared to brick walled houses, the mud-walled houses had less mosquitoes of all taxa, on any surface assessed. These differences varied but were significantly four times less for *An. funestus* (p = 0.01) (Table 4). Leaving walls un-plastered was also associated with greater *Anopheles* density on the walls, significantly more so with *An. funestus*. This effect was less evident when considering mosquitoes collected from roofs or other surfaces. Similarly, leaving the eave spaces open was associated with higher vector densities on the walls and other surfaces, but not on roofs. Finally, there were more mosquitoes on walls below one metre. Full details are provided in Table 4.

**Table 4:** Relationship between of household risk factors and indoor temperatures on mosquito resting preference on different surfaces

		*Anopheles funestus*		*Anopheles arabiensis*		*Culex* mosquitoes	
		RR (95% CI)	p value	RR (95% CI)	p value	RR (95% CI)	p value
Number of mosquitoes caught resting on walls							
Roof type	Iron sheets	1.00		1.00		1.00	
	Grass thatch	2.20 (0.87–5.56)	0.090	1.93 (0.46–8.10)	0.400	1.04 (0.43–2.46)	0.940
Wall type	Brick	1.00		1.00		1.00	
	Mud	0.17 (0.07–0.41)	0.001	0.33 (0.09–1.24)	0.100	0.97 (0.42–2.26)	0.950
Interior walls	Plastered	1.00		1.00		1.00	
	Un-plastered	3.66 (1.34–10.02)	0.010	1.65 (0.22–12.25)	0.620	0.96 (0.38–2.48)	0.940
Eave space	Closed	1.00		1.00		1.00	
	Open	0.38 (0.13–1.13)	0.080	1.68 (0.23–12.26)	0.610	1.04 (0.40–2.74)	0.940
Increasing no. rooms		1.51 (1.08–2.11)	0.020	2.48 (1.38–4.47)	0.002	1.16 (0.86–1.58)	0.330
Increasing wall height		0.38 (0.13–1.11)	0.080	0.12 (0.02–0.79)	0.030	1.05 (0.42–2.65)	0.910
Increasing max. temp.		0.97 (0.93–1.02)	0.220	1.16 (1.07–1.25)	0.001	1.02 (0.97–1.07)	0.410
Number of mosquitoes caught resting on the underside of roofs							
Roof types	Iron sheet	1.00		1.00		1.00	
	Grass thatch	6.07 (1.78–20.70)	0.004	92.16 (9.90–857.94)	0.001	3.96 (1.51–10.36)	0.005
Wall type	Brick	1.00		1.00		1.00	
	Mud	0.27 (0.09–0.77)	0.010	0.39 (0.09–1.68)	0.210	0.80 (0.32–2.01)	0.630
Interior walls	Plastered	1.00		1.00		1.00	
	Un-plastered	1.55 (0.44–5.46)	0.500	0.29 (0.02–4.74)	0.390	0.74 (0.26–2.12)	0.570
Eave space	Closed	1.00		1.00		1.00	
	Open	0.25 (0.06–0.98)	0.046	0.91 (0.06–14.53)	0.910	0.53 (0.18–1.54)	0.240
Increasing no. rooms		1.48 (0.97–2.26)	0.070	2.49 (1.25–4.96)	0.010	1.44 (1.03–2.00)	0.030
Increasing wall height		0.25 (0.06–0.95)	0.040	0.55 (0.06–4.71)	0.570	0.87 (0.31–2.41)	0.790
Increasing max. temp.		1.02 (0.97–1.07)	0.410	1.13 (1.06–1.20)	0.001	0.98 (0.93–1.02)	0.300
Number of mosquitoes caught resting on other surfaces inside the houses							
Roof types	Iron sheet	1.00		1.00		1.00	
	Grass thatch	2.12 (0.85–5.31)	0.110	3.75 (0.88–16.03)	0.070	1.66 (0.68–4.02)	0.260
Wall type	Brick	1.00		1.00		1.00	
	Mud	0.22 (0.09–0.55)	0.001	0.59 (0.13–2.78)	0.510	0.92 (0.38–2.21)	0.840
Interior walls	Plastered	1.00		1.00		1.00	
	Un-plastered	0.92 (0.33–2.54)	0.870	2.77 (0.44–17.51)	0.280	0.81 (0.30–2.13)	0.660
Eave space	Closed	1.00		1.00		1.00	
	Open	2.91 (1.00–8.46)	0.049	1.95 (0.28–13.71)	0.500	1.23 (0.46–3.33)	0.680
Increasing No. rooms		1.16 (0.84–1.60)	0.370	1.61 (0.90–2.88)	0.110	1.25 (0.91–1.71)	0.160
Increasing wall height		0.84 (0.29–2.41)	0.750	2.68 (0.46–15.65)	0.270	3.18 (1.21–8.33)	0.020
Increasing max. temp.		0.93 (0.90–0.97)	0.001	1.12 (1.06–1.19)	0.001	1.03 (0.98–1.08)	0.280

### *Anopheles* sibling species and *Plasmodium* infections

A subsample of 191 *An. gambiae* s.l. and 623 *An. funestus* s.l. were assayed for identification of sibling species, and presence of infectious stages of *P. falciparum*, i.e. sporozoites in the salivary glands. In the *An. gambiae* s.l. samples, there was an overall PCR amplification of 93.2% (n = 178), of which 100% were *An. arabiensis,* and none had sporozoite infections. For *An. funestus* s.l., PCR amplification was 89.1% (n = 555), of which 93.1% were *An. funestus* sensu stricto (s.s.) (n = 517), and 6.8% were *Anopheles rivulorum* (n = 38). None of the *An. rivolurum,* nor the un-amplified samples had sporozoites infections, but four of the *An. funestus* s.s. were sporozoite positive (0.8%).

### Mosquito blood meal sources and parity statuses

Based on the blood-meal ELISA assays done on 45 blood-fed *An. arabiensis*, more than half had human blood (55.56%; n = 25). The rest had blood from cattle (20%; n = 9), dogs (15.6%; n = 7), chickens (2.2%; n = 1) as well as mixed blood from dogs and cattle (4.4%; n = 2) and from humans and dogs (2.2%; n = 1). For *An. funestus* s.s., 224 blood-fed females were tested, the majority of which had obtained blood from humans (90.6%; n = 203). The rest of the *An. funestus* had blood from chicken (2.2%; n = 5), cattle (1.8%; n = 4), dog (0.9%; n = 2), mixtures of human and cattle blood (2.7%; n = 6) or human and chicken blood (1.8%; n = 4). Lastly, for *An. rivulorum,* only seven samples were tested, six of which had human blood in their guts (85.7%), the other having fed on cattle (14.3%). Of 67 *An. arabiensis* dissected, 53.7% (n = 36) were parous and 46.3% (n = 31) were nulliparous. While of 160 *An. funestus* dissected, only 36.9% (n = 59) were parous and the rest were nulliparous.

## Discussion

This research investigated the resting behaviours of malaria mosquitoes inside typical house types in rural south-eastern Tanzanian villages where *An. arabiensis* and *An. funestus* are the main vectors, the latter contributing more than 80% of all cases [44]. The main finding was that consistently less than one-third of mosquitoes that enter houses typically rest on walls, which are the main target for IRS campaigns. In fact, significant proportions regularly rest on surfaces other than walls or roofs (which are also sometime sprayed). These other surfaces include household items such as furniture, utensils, clothing and also on floors, places that are rarely sprayed. As historically observed [39, 40], this current study determined that malaria vectors do not rest only on walls, where they can be targeted with IRS. Instead, all surfaces inside houses are potential resting site for mosquitoes. The majority of *An. funestus* and *An. arabiensis* rest on surfaces other than walls, such as on the underside of roofs, bed nets, floors, furniture, utensils and clothes. However, variations were observed between vector species and house designs. In addition, assessment of interaction between resting surfaces and time of collection suggested that the time of collection had little to no effect on resting preference of mosquitoes (Additional file 1).

Indoor residual spraying (IRS) and long-lasting insecticide-treated nets (LLINs), despite having been tremendously impactful [2], are now perceived as inadequate for the goal of malaria elimination [59–61], partly due to the rise of insecticide resistance [10, 11] and changes in mosquito biting behaviours [12–14]. These challenges may result from, and can be compounded by extensive and improper implementation of the insecticide based strategies [16–18]. For example, incomplete coverage of all mosquito resting surfaces with IRS inside houses could lead to lower coverage of indoor surfaces with insecticides, sub-optimal dosing of the mosquitoes and hence reduced communal impact of the interventions. Therefore, to attain malaria elimination targets, current interventions need improvements to maximize effectiveness. This requires extensive understanding of mosquito behaviours inside houses, and how these mosquitoes would respond to indoor interventions, notably IRS and ITNs.

The composition of indoor resting mosquitoes observed in this study was of fairly different physiological ages and few infectious *Anopheles*. Also, the bloodmeal sources suggest that even outdoor biting mosquitoes rested indoor. This study, therefore, suggests that expanding target surfaces inside houses when spraying insecticides would increase impact of IRS on mosquito populations. Where this is not possible, a behaviour change communication programme can be implemented to sensitize and educate people on dangers of mosquitoes resting indoors on surfaces such as hanging clothes and darkened surfaces inside the homes. The study will also enable implementers to select the most important surfaces for IRS, in cases where resources are limited. IRS campaigns usually involve removal of household items before spraying is conducted [23]. However, once these items are returned to the houses, they form important resting surfaces free of insecticides. Since the study involved multiple collections at different times of day and night, the observed resting patterns are likely the natural patterns. It is however unclear whether there are any frequent movements of mosquitoes between resting surfaces, and how such movements may influence overall impact of IRS.

More importantly, these findings highlight specific gaps and limitations of IRS, and the need for more comprehensive interventions such as house improvement. As an example, house screening would not be affected by mosquito resting behaviours but would instead reduce overall densities in the homes. Another way would be to expand, as much as possible, the IRS targeted surfaces to include undersides of roofs and other sprayable surfaces (such as underneath beds, tables and other furniture) to have increased impact on the mosquitoes. Thirdly, coupling IRS with strategies to minimize mosquito resting on non-sprayable surfaces might also enhance impact. Such strategies may include, but are not limited to proper storage of household items, e.g. by placing these items inside enclosures such as cupboards. This could reduce potential surfaces for mosquito to rest, which may maximize mosquito contacts with treated surfaces. Without considering surfaces other than walls, our current efforts, targeting mosquito vectors with IRS might limit the impact of IRS on elimination and control outcomes. However, it is also recognized that proportion of mosquitoes resting on surfaces other than walls does not mean that these mosquitoes would never come into contact with walls or would not be killed by IRS. For this reason, additional studies may be required to examine how these differential resting behaviours and localization of the vectors in different house types actually impact effectiveness of IRS.

Indoor residual spraying remains one of the mainstays of malaria control in Africa, and is widely popular despite high costs. It is currently promoted in Africa mostly through the US Presidents Malaria Initiative [27] and national programmes often alongside LLINs, but was historically the most dominant tool in Africa and elsewhere starting from the Global Malaria Eradication period [21, 22]. It has indeed been associated with major reductions in malaria cases in the southern Africa region in past decades [62, 63], and remains an important component of their malaria control arsenal. The spraying procedures are generally standardized to achieve scale and reduce costs [23], and generally target walls and ceilings occasionally where these exist. As a result, the spraying operations may not adequately capture the full-spectrum of resting spaces used by malaria vectors or others.

The findings of this current study are in line with previous studies on resting preference of *Anopheles* mosquitoes inside houses [40]. However, this study extended the mosquito collections to cover more potential sites inside human inhabited dwellings, and also examined differences between different house types. It also described relationships between house designs and microclimate, with resting preferences of the *An. funestus*, *An. arabiensis,* and *Culex* mosquitoes. For example, grass thatched roofs were associated with higher proportions of *An. funestus* on roofs. When houses had open eaves, proportion of *An. funestus* increased on other surfaces, but increase in indoor maximum temperature was associated with decrease in proportion of *An. funestus* on other surfaces.

Insecticide resistance has led to shift of insecticides used in IRS to non-pyrethroid insecticides such as pirimiphos-methyl and neonicotinoids [1, 10]. However, recently a countrywide survey in Tanzania detected resistance against pirimiphos-methyl in several sites within the country [64]. The current IRS practices clearly miss several surfaces where mosquitoes rest, a situation, which could exacerbate the challenge of insecticide resistance and further compromise IRS. As mentioned earlier in this paper, understanding the resting behaviours of malaria vectors is crucial, if at all IRS is going to be widely used in malaria endemic countries including Tanzania. The gaps identified in this study can be compounded by insecticide resistance, and therefore need urgent attention to ensured effectiveness.

Though mostly successful, this study also had a few limitations. First, most collections of mosquitoes were done in the morning, when people were active participating in household chores. This might have influenced the choice of mosquitoes on resting surfaces during the day. Collections during the day might also have underestimated mosquitoes resting on surfaces such as floors and utensils. Second, the type and number of possessions inside houses are related to house types, since both are linked to wealthy/income. Mud houses are unlikely to have bigger furniture and rarely items inside these houses are properly arranged. It is likely that resting patterns of mosquitoes between individual house type was influenced by type and number of surfaces inside houses. Thus, influencing observed differences in resting preference among house types involved. Unfortunately, this phenomenon was not assessed in this study. Third, this study was conducted in villages which are not protected with IRS. However, mosquitoes have been shown in multiple studies to change their behaviours with interventions. Therefore, it is important that future studies should be carried-out to assess indoor resting preference of mosquitoes in houses protected with IRS.

## Conclusion

This study has demonstrated that while IRS typically uses contact insecticides against adult mosquitoes on walls, and occasionally roofs and ceilings, significant proportions of malaria vectors rest on other surfaces not usually sprayed during IRS campaigns. The study also demonstrates that the spraying gaps are influenced by house designs. For example, in grass-thatched houses, up to one-third of mosquitoes consistently rest on surfaces other than walls or roofs, are therefore not effectively controlled by contact insecticides. These gaps can reach two-thirds of mosquitoes in metal-roofed houses. It remains unclear how the observed mosquito habits could impact overall effectiveness of IRS. However, there is need to incorporate locally-obtained data on mosquito resting behaviours to maximize potential of IRS. Besides, other interventions such as improved housing should be prioritized to more comprehensively tackle indoor-biting and indoor-resting mosquitoes. Expanding IRS targeted surfaces inside houses can also be impactful. However, given the costs of IRS and logistical challenges associated with spraying non-standard surfaces, this approach in resource limited settings may not sustainable.

## Supplementary information

**Additional file 1: Figure S1.** Overall densities of *Culex* mosquitoes, from different resting surfaces in houses. This data is aggregated for all house types in the study area. Estimation plots are provided to depict distribution of residuals between collections from roofs or other surfaces, and the collections on walls. The number of mosquitoes resting on either roofs or other surfaces are considered significantly higher or lower based on how far the means of the residuals are above or below the reference line. **Figure S2.** Comparison of densities of *Culex* mosquitoes, from different resting surfaces in different house types. Estimation plots are provided to depict distribution of residuals between collections from roofs or other surfaces, and the collections on walls. The number of mosquitoes resting on either roofs or other surfaces are considered significantly higher or lower based on how far the means of the residuals are above or below the reference line. **Table S1.** Summary statistics of interactions between mosquitoes of different species collected from different resting surfaces (walls, roofs and other surfaces) and time.

## Data Availability

The datasets used and/or analysed during the current study are available from the corresponding author upon reasonable request.

## Abbreviations

ACT: artemisinin-based combination therapy
AIC: Akaike information criterion
ELISA: enzyme-linked immunosorbent assay
GLMM: generalized linear mixed effects model
HHMI: Howard Hughes Medical Institute
IHI: Ifakara Health institute
IRS: indoor residual spraying
ITN: insecticide-treated net
LLIN: long-lasting insecticide-treated net
PCR: polymerase chain reaction
PMI: President’s Malaria Initiative
RR: relative rate ate ratio
WHO: World Health Organization
