# Supplementary material for: Preferred resting surfaces of dominant malaria vectors inside different house types in rural south-eastern Tanzania
Source: Malar J. 2020 Jan 15;19:22. doi: 10.1186/s12936-020-3108-0 (PMC6964015; doi:10.1186/s12936-020-3108-0)
Supplement: Supplementary file 1 — Additional file 1: Figure S1. Overall densities of Culex mosquitoes, from different resting surfaces in houses. This data is aggregated for all house types in the study area. Estimation plots are provided to depict distribution of residuals between collections from roofs or other surfaces, and the collections on walls. The number of mosquitoes resting on either roofs or other surfaces are considered significantly higher or lower based on how far the means of the residuals are above or below the reference line. Figure S2. Comparison of densities of Culex mosquitoes, from different resting surfaces in different house types. Estimation plots are provided to depict distribution of residuals between collections from roofs or other surfaces, and the collections on walls. The number of mosquitoes resting on either roofs or other surfaces are considered significantly higher or lower based on how far the means of the residuals are above or below the reference line. Table S1. Summary statistics of interactions between mosquitoes of different species collected from different resting surfaces (walls, roofs and other surfaces) and time. [file 12936_2020_3108_MOESM1_ESM.docx]

**Additional material**


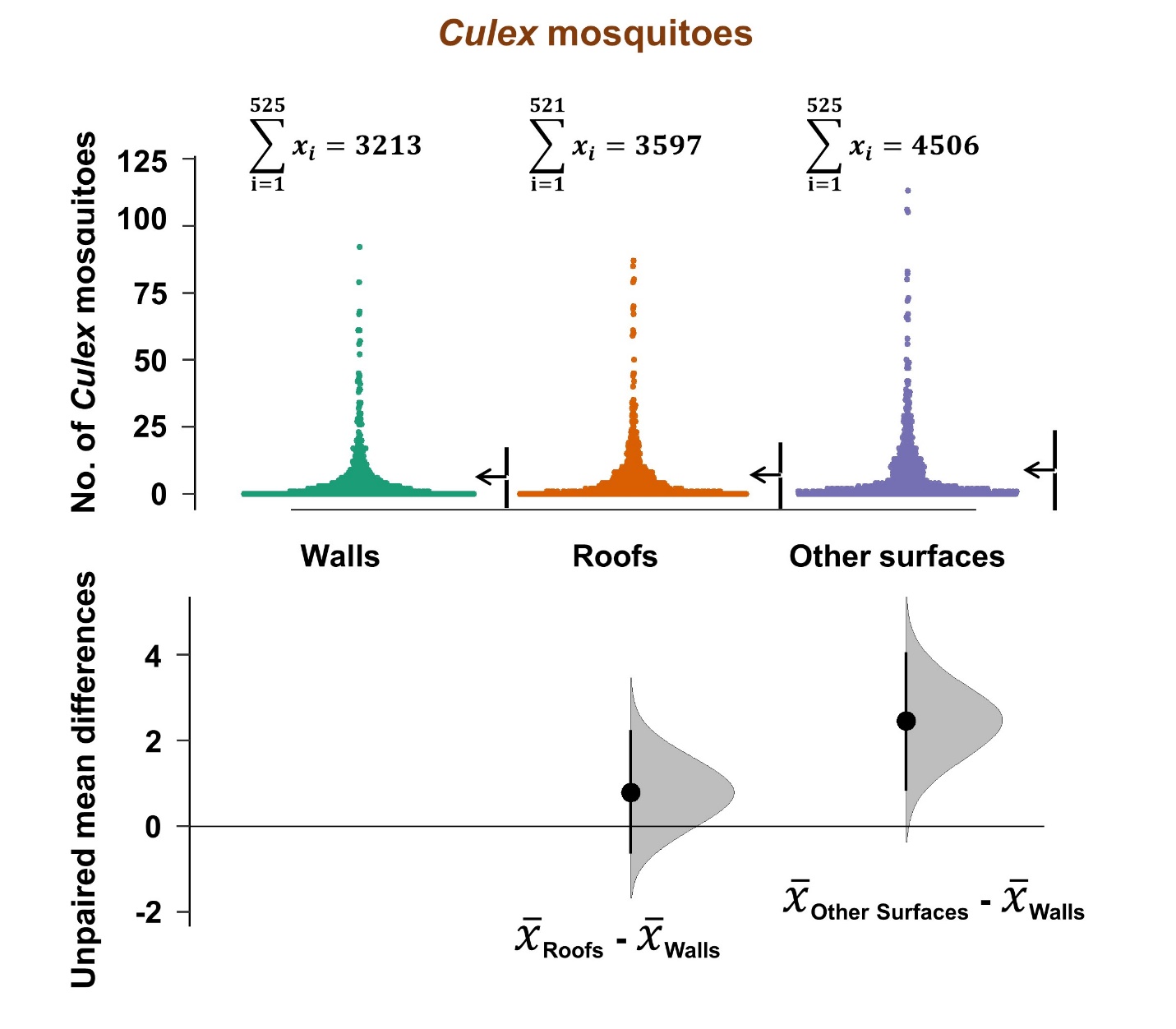


**Figure S1.** Overall densities of *Culex* mosquitoes, from different resting surfaces in houses. This data is aggregated for all house types in the study area. Estimation plots are provided to depict distribution of residuals between collections from roofs or other surfaces, and the collections on walls. The number of mosquitoes resting on either roofs or other surfaces are considered significantly higher or lower based on how far the means of the residuals are above or below the reference line.


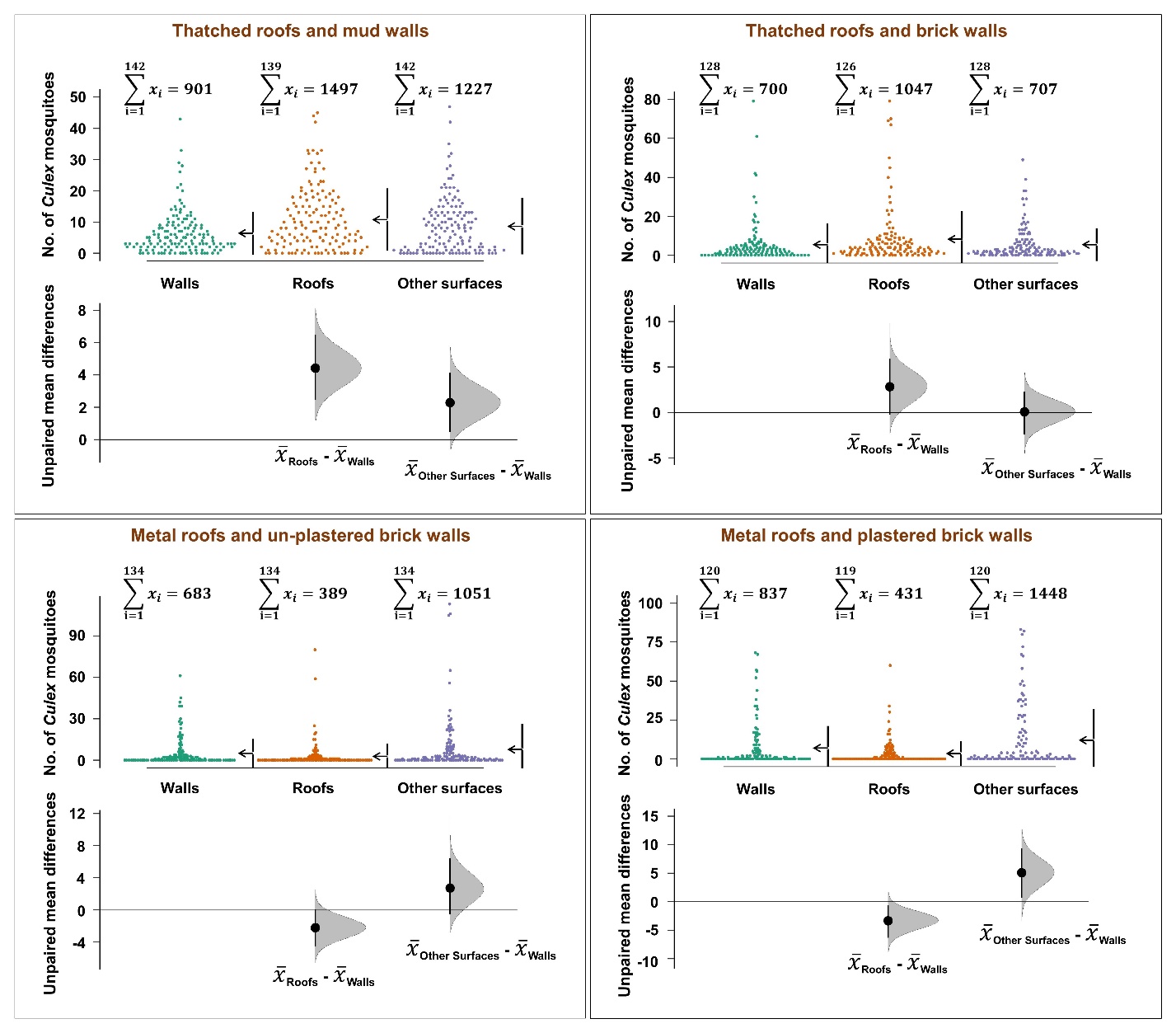


**Figure S2.** Comparison of densities of *Culex* mosquitoes, from different resting surfaces in different house types. Estimation plots are provided to depict distribution of residuals between collections from roofs or other surfaces, and the collections on walls. The number of mosquitoes resting on either roofs or other surfaces are considered significantly higher or lower based on how far the means of the residuals are above or below the reference line.

**Table S1.** Summary statistics of interactions between mosquitoes of different species collected from different resting surfaces (walls, roofs and other surfaces) and time

|  | *An. funestus* | |  | *An. gambiae* | |  | *Culex* mosquitoes | |
| --- | --- | --- | --- | --- | --- | --- | --- | --- |
|  | RR (95% CI) | p value |  | RR (95% CI) | p value |  | RR (95% CI) | p value |
| Walls | 1 |  |  | 1 |  |  | 1 |  |
| Roofs | 1.06 (0.77-1.46) | 0.728 |  | 1.81 (0.92-3.57) | 0.087 |  | 1.60 (1.17-2.19) | 0.003 |
| Other surfaces | 1.64 (1.20-2.23) | 0.002 |  | 1.95 (0.99-3.81) | 0.053 |  | 1.61 (1.18-2.20) | 0.003 |
| Morning (6a.m. and 9a.m.) | 0.52 (0.20-1.41) | 0.201 |  | 0.73 (0.13-4.10) | 0.716 |  | 0.67 (0.30-1.50) | 0.330 |
| Morning (9a.m. and 12p.m.) | 0.57 (0.21-1.58) | 0.281 |  | 0.43 (0.08-2.46) | 0.342 |  | 0.57 (0.25-1.30) | 0.183 |
| Evening (6p.m. and 8p.m.) | 0.74 (0.53-1.05) | 0.093 |  | 2.57 (1.29-5.13) | 0.007 |  | 1.25 (0.89-1.76) | 0.203 |
| Night (6p.m. and 8p.m.) | 1.21 (0.88-1.65) | 0.242 |  | 1.62 (0.80-3.27) | 0.177 |  | 1.19 (0.86-1.64) | 0.306 |
| Roofs * Morning (6a.m. and 9a.m.) | 1.07 (0.69-1.64) | 0.769 |  | 1.06 (0.45-2.51) | 0.898 |  | 0.79 (0.54-1.15) | 0.213 |
| Other surfaces * Morning (6a.m. and 9a.m.) | 0.96 (0.63-1.45) | 0.837 |  | 1.54 (0.66-3.63) | 0.321 |  | 0.82 (0.56-1.19) | 0.292 |
| Roofs * Morning (9a.m. and 12p.m.) | 0.77 (0.44-1.35) | 0.369 |  | 1.49 (0.52-4.25) | 0.461 |  | 0.65 (0.42-1.00) | 0.051 |
| Other surfaces * Morning (9a.m. and 12p.m.) | 0.89 (0.53-1.52) | 0.679 |  | 1.11 (0.38-3.25) | 0.846 |  | 0.95(0.62-1.46) | 0.828 |
| Roofs * Evening (6p.m. and 8p.m.) | 1.72 (1.09-2.72) | 0.020 |  | 0.96 (0.40-2.31) | 0.931 |  | 0.76 (0.48-1.19) | 0.227 |
| Other surfaces * Evening (6p.m. and 8p.m.) | 0.92 (0.58-1.46) | 0.730 |  | 0.86 (0.36-2.07) | 0.744 |  | 0.90 (0.57-1.42) | 0.655 |
| Roofs * Night (6p.m. and 8p.m.) | 1.07 (0.69-1.66) | 0.759 |  | 1.01 (0.41-2.53) | 0.975 |  | 0.76 (0.49-1.18) | 0.223 |
| Other surfaces * Night (6p.m. and 8p.m.) | 0.91 (0.59-1.39) | 0.650 |  | 1.36 (0.56-3.35) | 0.498 |  | 0.95 (0.61-1.47) | 0.806 |
